# PreFold-dG: estimating binding affinity of protein–protein interaction from intermediate representations of protein folding model

**DOI:** 10.1093/bioinformatics/btag489

**Published:** 2026-08-21

**Authors:** Sungjoon Park, Soorin Yim, Dongyun Kim, Kiwoong Yoo, Doyeong Hwang, Kyungwook Lee, Jongseong Jang, Kiyoung Kim

**Affiliations:** Bio Intelligence Lab, LG AI Research, Seoul, 07789, Republic of Korea; Bio Intelligence Lab, LG AI Research, Seoul, 07789, Republic of Korea; Bio Intelligence Lab, LG AI Research, Seoul, 07789, Republic of Korea; School of Biological Science, Seoul National University, Seoul, 08826, Republic of Korea; Bio Intelligence Lab, LG AI Research, Seoul, 07789, Republic of Korea; Bio Intelligence Lab, LG AI Research, Seoul, 07789, Republic of Korea; Bio Intelligence Lab, LG AI Research, Seoul, 07789, Republic of Korea; Bio Intelligence Lab, LG AI Research, Seoul, 07789, Republic of Korea; Bio Intelligence Lab, LG AI Research, Seoul, 07789, Republic of Korea

## Abstract

**Motivation:**

Binding affinity governs how proteins interact and underlies essential biological processes. Computational approaches have been developed to simulate and predict protein binding, but the scarcity of high-quality data has imposed significant constraints. One consequence is that most methods focus on predicting mutational changes in binding affinity (ΔΔG), rather than binding affinity (ΔG) itself. This practice risks overfitting to skewed data distributions, limiting the generalizability of predictions. Recent advances in protein structure prediction have enabled computational modeling of protein conformations in mass, providing rich structural information from which binding interactions can be largely explained. However, leveraging these advances for effective prediction of binding affinity has yet to translate into reliable predictions.

**Results:**

We present PreFold-dG, a model that estimates binding affinities of protein complexes utilizing intermediate embeddings from Boltz-2, an open-source foundation model for protein structure prediction. Our approach aggregates residue-level information weighted by interresidue distance, and predicts ΔG directly rather than its derivative, ΔΔG. PreFold-dG achieved state-of-the-art performance on well-established binding affinity prediction benchmarks and demonstrated robustness on independent test sets. Ablation studies suggest that all intermediate embeddings are utilized in the prediction, whereas their contributions to modeling ΔΔG and ΔG vary. We further validated our model through case studies on real-world broadly neutralizing antibody data with evolutionary relevance.

**Availability:**

https://github.com/LGAI-Research/PreFold-dG.

## 1 Introduction

Protein-protein interactions (PPIs) play a central role in cellular processes. Quantitative characterization of PPI binding affinity is thus important for both fundamental understanding of molecular biology and practical applications such as drug discovery and protein engineering. Classical approaches to binding affinity estimation rely on experimentally resolved crystal structures and physics-based scoring functions such as FoldX (Delgado *et al.* 2019) or Rosetta Flex-ddG (Barlow *et al.* 2018). While physically grounded, these methods are often computationally expensive and limited in predictive accuracy. Recent advances in protein structure prediction, most notably AlphaFold3 (Abramson *et al.* 2024), have enabled accurate modeling of protein structures at large scale. A growing body of data-driven models has since leveraged these predicted structures, along with sequences or protein language models, to estimate binding affinity. Despite this progress, several fundamental limitations remain in current methodologies.

Most existing models predict changes in binding affinity upon mutation (ΔΔG) rather than the absolute binding free energy (ΔG) itself. This convention reflects the substantial variability of absolute ΔG measurements, which arise from differences in biophysical assays and procedures across laboratories (Sanavia *et al.* 2020). ΔΔG is by definition a differential quantity derived from paired ΔG values, and mapping mutant and wild-type complex pairs directly to a scalar through black-box transformations without explicit ΔG estimation sacrifices arithmetic consistency and explainability. Furthermore, the entangled paired-input architecture limits the training data to samples for which both wild-type and mutant measurements are jointly available, excluding the large body of individual ΔG measurements. Given the skewness of the ΔΔG distribution in benchmark datasets, this data constraint may limit the model’s capacity to learn the broader landscape of binding affinities. More broadly, because these models require wild-type reference as input, they are inherently inapplicable to de novo design scenarios where no such baseline exists.

In this work, we propose two key ideas to address these limitations. First, we reformulate the ΔΔG prediction problem as to its natural definition: the difference between ΔG values of mutant and wild-type, each predicted by the same module. This decomposition guarantees anti-symmetry and transitivity by construction, and makes each ΔG prediction individually inspectable (Montanucci *et al.* 2022). To address the variability of absolute ΔG measurements, we devised a normalization-based relabeling that mitigates cross-study inconsistencies.

Second, instead of relying on the final predicted coordinates of structure prediction models, we use the learned intermediate representations of Boltz-2 (Passaro *et al.* 2025), one of the first open-source implementations of AlphaFold3, as ingredients for ΔG estimation. Protein complexes are inherently dynamic. A single set of predicted coordinates captures only one snapshot of the bound-state ensemble. The intermediate representations, by contrast, have not yet been collapsed into a single structural output, and may retain richer information about residue interactions than the final coordinates alone. The efforts to model binding affinity using confidence metrics of protein structures (Nori *et al.* 2025), or more directly, to infer an ensembled conformation to predict multi-state representations (Kalakoti and Wallner 2025), also concur with this view.

Our approach achieves state-of-the-art (SOTA) performance across benchmarks and independent test sets with robust generalization. This formulation also enables independent ΔG prediction, which may facilitate broader data utilization and de novo applications in future work. We further provide mechanistic analyses and case studies illustrating its practical utility.

## 2 Methods

### 2.1 Problem formulation

The binding affinity of a protein complex is quantified by the change in Gibbs free energy (ΔG) between bound and unbound states, $\Delta G=G_{\text{bound}}-G_{\text{unbound}}$, where negative values indicate spontaneous binding. Given wild-type and mutant complexes, the change in binding affinity upon mutation is defined as:


$$\begin{array}{c} \Delta G_{\text{mut}}=G_{\text{mut},\text{bound}}-G_{\text{mut},\text{unbound}}\\ \Delta G_{\text{wt}}=G_{\text{wt},\text{bound}}-G_{\text{wt},\text{unbound}}\\ \Delta \Delta G=\Delta G_{\text{mut}}-\Delta G_{\text{wt}} \end{array}$$

This formulation cancels method-specific biases and directly indicates whether a mutation is favorable or unfavorable for binding. Accordingly, most existing computational approaches formulate binding affinity prediction as a paired-input problem:


f:(mutant,wild-type)→ΔΔG

However, this formulation entangles the mutant and wild-type representations within black-box models. In this work, we instead decompose ΔΔG prediction into independent estimation of absolute binding free energies:


h:(protein complex)→ΔG

ΔΔG=h(mutant)−h(wild-type)

This decomposition guarantees two desirable mathematical properties. *Anti-symmetry*: $\Delta \Delta G_{\text{wt}\to \text{mut}}=-\Delta \Delta G_{\text{mut}\to \text{wt}}$, that is, reversing the direction of mutation exactly negates the predicted value. *Transitivity*: $\Delta \Delta G_{A\to C}=\Delta \Delta G_{A\to B}+\Delta \Delta G_{B\to C}$, that is, the prediction is independent of the path taken through sequence space. Both follow from computing ΔΔG as the difference of two ΔG values. Conventional architectures do not automatically guarantee either property without additional architectural constraints (Deng *et al.* 2025). Moreover, this decomposition enables intermediate ΔG predictions and their associated embeddings individually inspectable, adding layer of transparency and interpretability to the model’s behavior.

### 2.2 Datasets

SKEMPI 2.0 (Jankauskaitė *et al.* 2019) is the most widely used benchmark for binding affinity prediction upon mutation. The dataset contains 7085 manually curated ΔΔG measurements spanning 345 PDBs, covering single- and multi-point mutations of diverse protein complexes. Binding affinities were measured with various biophysical assays (e.g. SPR, ITC, ELISA). SKEMPI contains duplicate entries and cross-study measurement variability, which we address through the pre-processing steps in Section 2.3.

For an independent evaluation, we use HER2 binder dataset adopted by GearBind (Cai *et al.* 2024) from experimental measurements (Shanehsazzadeh *et al.* 2023). This dataset contains 417 mutations on antibody variants targeting HER2, all derived from the 1N8Z complex. Although this PDB entry also appears in SKEMPI, the two datasets differ substantially in character. SKEMPI predominantly contains single-point alanine substitutions, whereas the HER2 dataset includes diverse multi-point mutations with a distinct ΔΔG distribution. Thus, it works as an out-of-distribution test set for assessing the generalizability of models trained on SKEMPI.

### 2.3 Pre-processing

SKEMPI contains duplicate entries from repeated measurements under different experimental conditions. We consolidate these by applying a sequential filter to each unique identifier, defined by the tuple (PDB, chains, mutations). We first retain entries from the latest dataset version, preferring SKEMPI v2.0 over v1.0. We rank experimental methods into three reliability tiers (Table S1) and discard lower-tier entries when higher-tier data are available. When multiple measurements remain, we take their median to mitigate the effect of outliers.

Entries sharing the same complex but different mutations may have inconsistent wild-type ΔG values due to cross-study variability. To resolve this, we compute a consensus wild-type ΔG for each complex using the same filtering and median aggregation, then recalibrate each mutant ΔG by adding the reported ΔΔG to this consensus wild-type anchor:


$$\Delta G_{\text{mut}}=\Delta G_{\text{wt},\;\text{consensus}}+\Delta \Delta G_{\;\text{reported}}$$

This procedure leverages the greater reliability of ΔΔG over ΔG values, while establishing internally consistent ΔG labels across all entries in a PDB. After filtering and calibration, the final dataset contains 5817 entries across 334 unique complexes.

### 2.4 Model

As described in Section 2.1 our approach decomposes ΔΔG prediction into independent ΔG computations for the mutant and wild-type complexes, using the same model. For each input, we employ intermediate embeddings extracted from Boltz-2 (Passaro *et al.* 2025). These embeddings consist of four tensors: s_inputs, s, z, and distogram, extracted from a forward pass of Boltz-2 given a protein complex as input. Their semantic descriptions and dimensionalities are summarized in Table 1, where *L* denotes total number of residues of all chains ($L_{A}+L_{B}$) in the complex.

**Table 1:** Embedding tensors extracted from the Boltz-2 foundation model.

Data	Shape	Description
s_inputs	[*L* , 384]	Initial single residue representation
s	[*L* , 384]	Refined single residue representation
z	[*L, L*,128]	Pairwise residue spatial representation
distogram	[*L, L*,64]	Pairwise residue distance distribution

*L* denotes combined total length of proteins.

**Figure 1:**
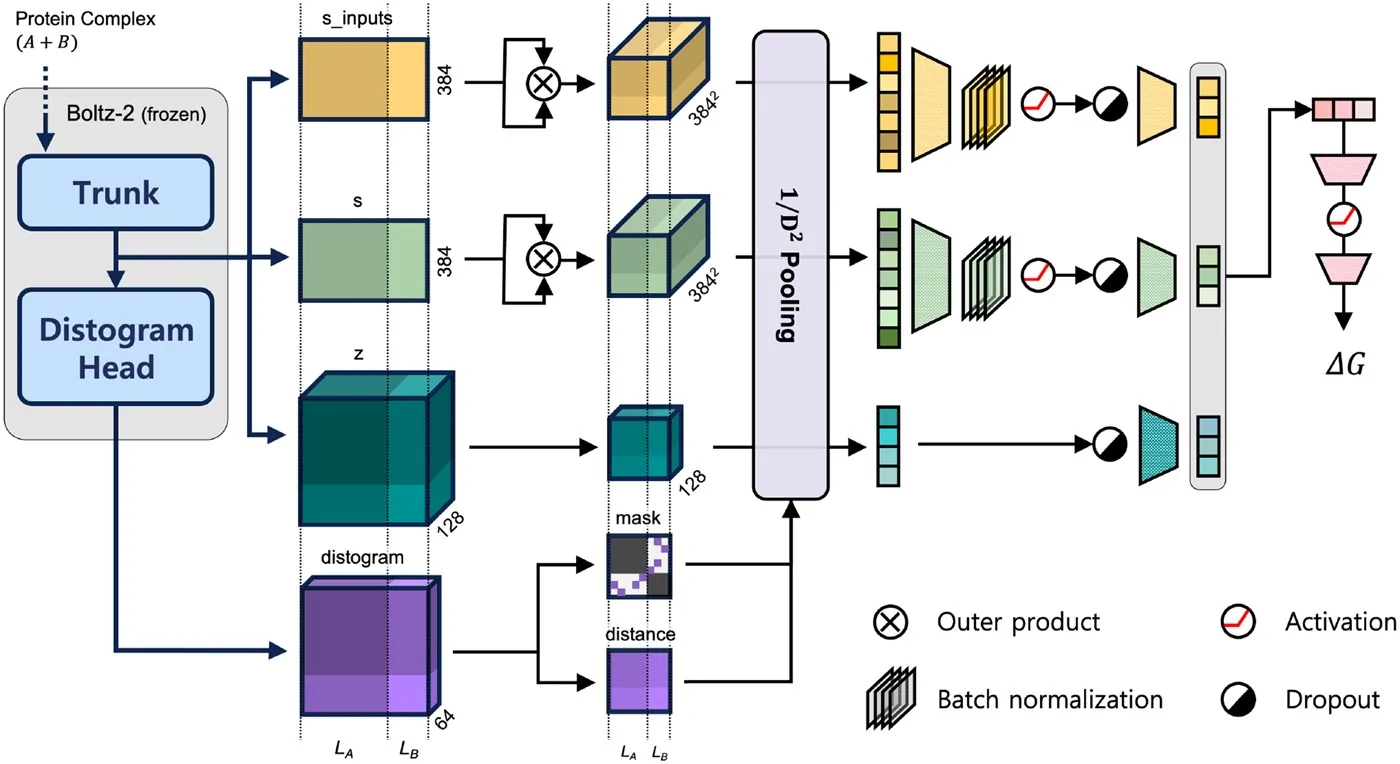
Overview of PreFold-dG. Proteins are encoded by frozen Boltz-2 into intermediate embeddings, which are passed to the downstream binding affinity prediction module. A single shared module independently predicts $\Delta G_{\text{mut}}$ and $\Delta G_{\text{wt}}$ from mutant and wild-type inputs, and ΔΔG is computed as their difference.

Figure 1 illustrates the architecture of our model, PreFold-dG. We hypothesize that binding affinity between two proteins is predominantly governed by interchain residue pairs, with their contribution modulated by spatial proximity. Given a protein complex (A+B), we first extract four embeddings from Boltz-2. These embeddings are defined over either individual residues (s_inputs, s) or residue pairs (z, distogram). To model binding affinities from an interchain residue-pair perspective, we compute the self outer product of first-order embeddings s_inputs and s along the residue axis following AlphaFold2 (Jumper *et al.* 2021), yielding tensors of shape [L,L,384×384].

Since different proteins comprise varying numbers of residues, the tensor dimensions differ by samples. To obtain fixed-size representations, we utilized distogram to perform distance-weighted pooling over s_inputs, s, and z. Concretely, we compute the expected distance from distogram and pool three embeddings with weights proportional to the inverse square of distance ($1/D^{2}$ Pooling), analogous to gravitational forces. Intrachain residue pairs are masked and assigned zero weights. After pooling, s_inputs and s are reduced to shape [384×384], and z to shape [128], irrespective of the original sequence length.

The pooled s_inputs and s tensors are subsequently projected to a lower-dimensional hidden space via a linear layer, followed by batch normalization, a nonlinear activation, and dropout, and z for only dropout. All three representations are then mapped to a shared embedding space of a same dimension. The three embeddings are integrated by averaging them into an aggregated embedding, which is then passed through a two-layer MLP prediction head to estimate ΔG. The change in binding free energy upon mutation, ΔΔG, is computed by subtracting the predicted ΔG of the wild-type complex from that of the mutant, both obtained by using an identical model.

Finally, the model is trained with a joint loss over $\Delta G_{\text{mut}}$, $\Delta G_{\text{wt}}$ and ΔΔG, combining mean squared error terms for all three objectives. Boltz-2 remains frozen throughout training, preserving the integrity of its embedding tensors independent of the downstream binding affinity prediction module. Detailed training configurations and hyperparameters used are provided in the Table S2.

## 3 Results

### 3.1 ΔΔG prediction

We evaluated PreFold-dG on SKEMPI under two different data splits, and on HER2. We report Pearson correlation coefficient (PCC), Spearman rank correlation coefficient (SRCC), root mean square error (RMSE), mean absolute error (MAE), and area under the ROC (AUROC). AUROC was measured by classifying binary signs of ΔΔG, thereby assessing whether the model correctly predicts the direction of mutational effect on binding affinity. Since overall correlation metrics can be inflated by coarse inter-PDB discrimination rather than fine-grained intra-PDB ranking, we additionally report macro-average per-structure PCC and SRCC, computed within each PDB group with at least 10 data points, consistently to prior studies. This more faithfully reflects models’ ability to rank mutations within the same protein complex (Wu and Li 2025), a property of direct relevance to applications such as affinity maturation.

#### 3.1.1 PDB-level split

A naive random split, in which mutations from the same PDB entry may appear in both training and test sets, yields inflated performance as the model can exploit structural similarity between training and test samples (Yu *et al.* 2025). To enforce separation at the complex level, Luo *et al.* (2023) proposed a 3-fold cross-validation scheme that partitions data such that all mutations belonging to the same PDB entry fall into the same fold. This split has been widely adopted and serves as the de facto standard benchmark. We compared PreFold-dG with existing methods under this protocol in Table 2 and Table S3. PreFold-dG outperforms existing methods on per-structure metrics, and achieves competitive performance on overall metrics. Most notably, per-structure metrics, which are more reliable for practical application (Luo *et al.* 2023), improve substantially. The decomposition (Section 2.1) explicitly disentangles per-complex baseline energy from mutational offsets. Given the limited mutational coverage and skewed PDB distribution in SKEMPI, models that fail to capture per-structure ΔΔG values are prone to poor generalization on unseen protein complexes (Wu *et al.* 2025).

**Table 2:** ΔΔG evaluation on the SKEMPI dataset using the 3-fold cross-validation split of RDE-Network (Luo *et al.* 2023).

Method	Per-structure		Overall				
	**PCC** ↑	**SRCC** ↑	**PCC** ↑	**SRCC** ↑	**RMSE** ↓	**MAE** ↓	**AUROC** ↑
FoldX (Delgado *et al.* 2019)	0.3789	0.3693	0.3120	0.4071	1.9080	1.3089	0.6582
Rosetta (Barlow *et al.* 2018)	0.3284	0.2988	0.3113	0.3468	1.6173	1.1311	0.6562
RDE-Network (Luo *et al.* 2023)	0.4448	0.4010	0.6447	0.5584	1.5799	1.1123	0.7454
PPIformer (Bushuiev *et al.* 2024)	0.4281	0.3995	0.6450	0.5304	1.6420	1.1186	0.7380
BA-DDG (Jiao *et al.* 2025)	0.5453	0.5134	0.7118	0.6346	1.4516	1.0151	0.7726
EBM-DDG (Soga *et al.* 2025)	0.5681	0.5184	0.7385	0.6516	1.3901	0.9871	**0.7941**
Light-DDG (Wu *et al.* 2025)	0.5440	0.5004	0.7429	**0.6767**	1.3837	0.9697	0.7935
Pythia-PPI (Tao *et al.* 2025)	0.5653	0.5271	**0.7850**	0.6365	**1.0796**	**0.7383**	0.7794
H3-DDG (Xu *et al.* 2026)	0.5686	0.5281	0.7501	0.6604	1.3665	0.9612	0.7920
PreFold-dG	**0.6109**	**0.5732**	0.7500	0.6597	1.6023	1.1292	0.7901

The best and second-best values are noted bold and underlined, respectively.

#### 3.1.2 Similarity-aware split

PDB-based separation does not account for cases where structurally identical or highly similar complexes are deposited under different PDB identifiers. Bushuiev *et al.* (2024) introduced a similarity-aware split that clusters complexes by interface structural similarity with five held-out proteins for validation, reducing cross-partition information leakage. PreFold-dG outperformed prior methods across most metrics, with improvements of +38% in SRCC and +46% in PCC (Table 3). Bushuiev *et al.* (2024) also noted that deep learning methods and physics-based ones such as Flex-ddG vary substantially in performance across different targets. PreFold-dG demonstrates the most balanced prediction across heterogeneous targets.

**Table 3:** ΔΔG evaluation on five held-out test proteins using the 3-fold cross-validation split of PPIformer (Bushuiev *et al.* 2024).

Method	**SRCC** ↑	**PCC ** ↑	**Precision ** ↑	**Recall ** ↑	**AUROC ** ↑	**MAE ** ↑	**RMSE ** ↓	**SRCC** ↑				
								Barnase	C3d	E6AP	YTM1	dHP1
Flex-ddG	0.55	0.57	**0.63**	0.62	0.84	**1.59**	2.00	0.82	0.68	0.29	**0.98**	−0.05
GEMME	0.38	0.41	0.60	0.49	0.74	2.16	2.81	0.40	0.61	0.20	0.79	−0.10
MSA transformer	0.31	0.36	0.51	0.38	0.70	6.13	6.93	0.43	0.61	0.37	0.05	0.09
ESM-IF	0.18	0.18	0.33	0.41	0.68	1.87	2.15	0.18	0.34	0.21	0.09	0.10
RDE-Network	0.24	0.30	0.54	0.65	0.67	1.70	2.02	0.58	0.68	0.21	0.15	−0.40
PPIformer	0.44	0.46	0.60	0.61	0.78	1.64	**1.94**	0.60	**0.75**	0.43	0.34	0.00
PreFold-dG	**0.76**	**0.83**	0.60	**0.75**	**0.85**	2.07	2.96	**0.87**	0.63	**0.44**	0.87	**0.40**

The best and second-best values are noted bold and underlined, respectively.

#### 3.1.3 Independent HER2 evaluation

To further evaluate PreFold-dG’s generalizability, we tested it on HER2 binders dataset following GearBind’s protocol (Cai *et al.* 2024). Multiple HER2-related complexes appear in SKEMPI, causing a potential information leakage. To mitigate this, we measured cross-dataset target homology using Smith–Waterman algorithm (Fig. S1). We identified and excluded nine HER2-containing PDB entries (Table S4) from training. The remaining complexes were split into five folds as in GearBind, and predictions are averaged across them. Table S5 and S6 show that PreFold-dG not only achieves SOTA performance but also exhibits the most stable scores across folds.

### 3.2 ΔG prediction

Our decomposition naturally yields per-complex ΔG estimates, a capability most ΔΔG models lack without fundamental architectural changes. To contextualize this, we selected BA-DDG (Jiao *et al.* 2025) as a baseline, as its modular architecture separately estimates ΔG for wild-type and mutant complexes, making it one of the rare ΔΔG models amenable to ΔG evaluation. We retrained BA-DDG with a joint loss over ΔΔG and ΔG, identical to our training protocol from Section 2.4.

Table 4 shows that PreFold-dG achieves strong prediction of ΔG in both overall and per-structure evaluations. BA-DDG also exhibits highly competitive prediction in overall, but shows degraded numbers in per-structure prediction. Also note that its ΔΔG prediction fell below the original model trained only with ΔΔG labels, from Table 2. This indicates that ΔG prediction poses challenges distinct from predicting ΔΔG, rather than being a corollary task that ΔΔG-oriented models can capture.

**Table 4:** ΔG evaluation on SKEMPI 3-fold cross-validation.

Obj.	Method	Per-structure		Overall		
		**PCC** ↑	**SRCC** ↑	**PCC** ↑	**SRCC** ↑	**RMSE** ↓
ΔG	BA-DDG	0.361	0.350	0.634	0.612	1.916
	PreFold-dG	0.611	0.573	0.711	0.712	1.719
ΔΔG	BA-DDG	0.408	0.393	0.682	0.553	1.697
	PreFold-dG	0.611	0.573	0.750	0.660	1.602

We observed a plausible explanation from juxtaposing per-structure predictions for ΔG and ΔΔG. The two should arithmetically be the same, since ranking orders are irrelevant to offset $\Delta G_{\text{wt}}$. However, BA-DDG uses ProteinMPNN as its backbone, whose decoding order for the wild-type residues is affected by given mutant pair. Consequently, BA-DDG implicitly encodes mutational loci and produces different $\Delta G_{\text{wt}}$ estimates every time, resulting in the gap. The degraded per-structure performance implies the potential risk of the entanglement of mutants and wild-types.

Reviewing Table 2, physics-based approaches such as FoldX and Rosetta Flex-ddG show minimal gaps between overall and per-structure correlation, naturally from their PDB-independent scoring. Deep learning models exhibit larger gaps, suggesting that some of their overall performance derives from inter-PDB discrimination rather than intrastructure resolution. PreFold-dG’s gap falls inbetween, retaining the representational capacity of a learned model while reducing reliance on inter-PDB shortcuts, an effect we attribute to the explicit ΔG decomposition that anchors predictions to individual complexes.

We additionally evaluated PreFold-dG on Adaptyv competition data (Cotet *et al.* 2025) across 24 targets with measured $K_{D}$ values for de novo designed binders in two settings: (i) binary classification of binders and non-binders, and (ii) regression on successful binders. Table S7 shows meaningful classification on the largest targets of EGFR (*n* = 768, AUROC = 0.69), NiV-G (*n* = 884, AUROC = 0.59), and PD-L1 (*n* = 75, AUROC = 0.72). This implies that PreFold-dG captures coarse binding signals from SKEMPI training to de novo designed sequences. However, it showed limited capability on the fine-grained ranking task, consistent with the reported difficulty of this task (Cotet *et al.* 2025). PreFold-dG’s standalone ΔG predictions transfer to de novo binders at a coarse level, but higher-resolution prediction requires further improvement.

### 3.3 Ablation studies

The degradation of BA-DDG under joint loss training suggests that the relabeling (Section 2.3) and joint loss function do not improve prediction on their own, but rather in synergy with specific architectural designs and intermediate embeddings. To isolate individual contributions, we conducted ablation studies for the features and the loss term (Table S8).

Ablating the data features s_inputs, s, and z all resulted in performance degradation, indicating that each captures a distinct aspect of binding affinity semantics, despite being an interconnected pipeline. Ablating z yielded the smallest drop, consistent with its 128-dimensional representation being primarily dedicated to inter-residue distance information used for structure construction. In contrast, ablating s produced the largest drop in ΔΔG prediction, while excluding s_inputs caused only a moderate drop. Notably, this trend does not hold for ΔG prediction, for which excluding s_inputs has the most deleterious effect. This observation aligns with our intuition that ΔG and ΔΔG depend on different aspects of the input representations. Modeling ΔΔG as a “delta” of ΔG can be an effective strategy, but only when the model captures features relevant to both tasks. Further discussion of the semantics of each embedding feature is provided in Section 3.4.

Ablating the loss components likewise led to performance drops across most metrics, with the largest degradation occurring on the task corresponding to the excluded supervision term. That is, removing each supervision term most strongly affected its directly associated task. However, supervising only on ΔG resulted in a less severe drop in ΔΔG prediction than the other way around, owing to their natural dependency. This supports our hypothesis that a model properly trained on ΔG labels can, in principle, predict ΔΔG labels, but not vice versa.

Since each wild-type structure is observed repeatedly alongside its mutant variants, the model may overfit to their representation. We investigated the sensitivity of PreFold-dG to the scale of $\Delta G_{\text{wt}}$ in the joint loss function. Table S9 shows that any downweighting strategy had no gain, at cost of degraded ΔG and per-structure ΔΔG prediction.

### 3.4 Intermediate embeddings analyses

**Figure 2:**
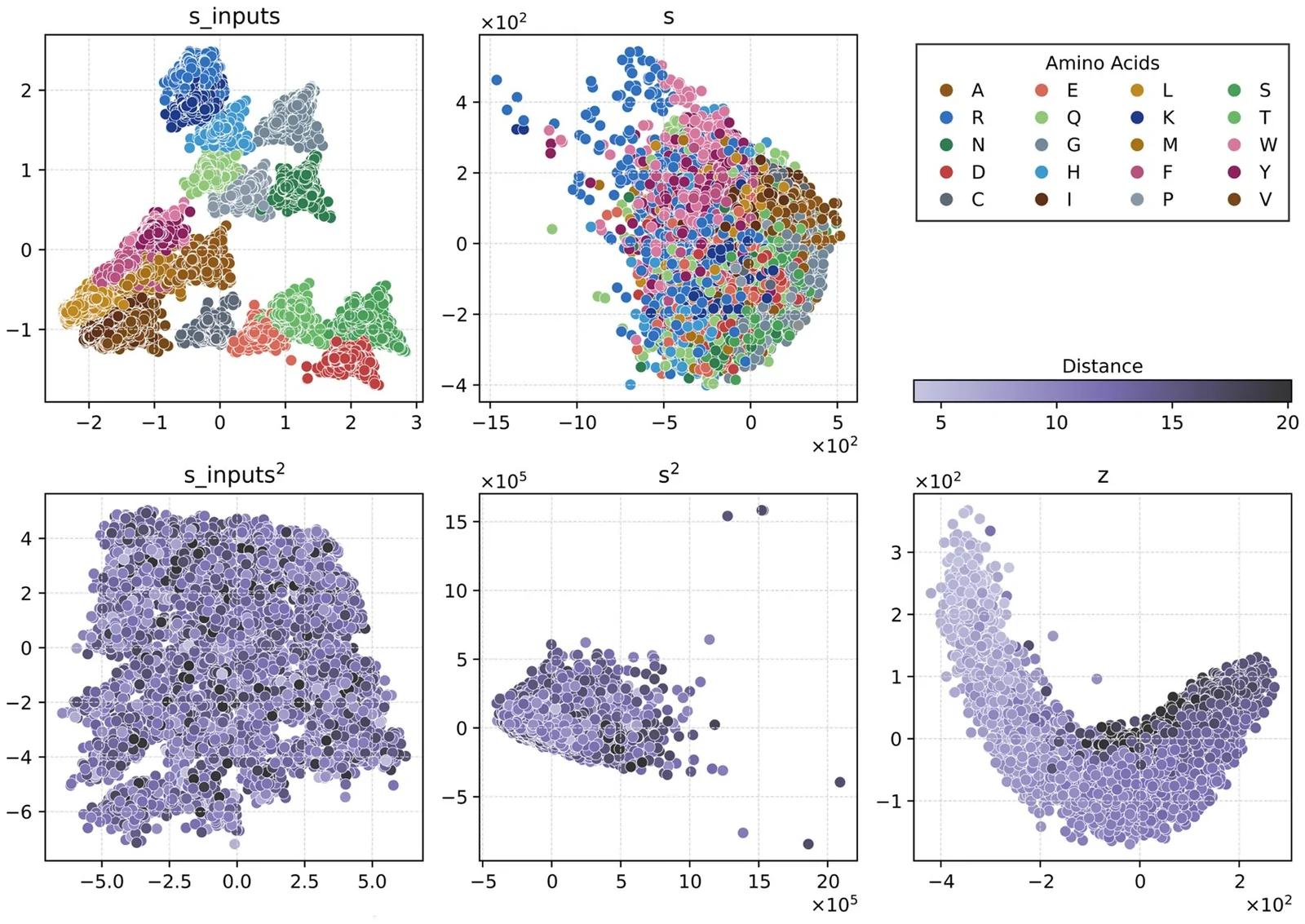
PCA of Boltz-2 intermediate embeddings. Self outer products of s_inputs and s are denoted $s\_\mathrm{input}s^{2}$ and $s^{2}$, respectively. Notable correlations are observed between s_inputs and amino acid type, and between z and interresidue distance.

To examine what information each feature encodes, we analyzed the intermediate embeddings from Boltz-2 plus the self outer products for the residue-level ones via PCA projection (Fig. 2). For computational efficiency and to ensure representation of diverse PDB groups, we only used wild-type protein complexes.

At residue level, s_inputs clusters by amino acid type, whereas s does not, despite its substantially larger magnitude and variance. This suggests that the dominant axes of variation in s capture spatial rather than sequence-level information. In analogy to natural language processing, s_inputs resembles a static word embedding, while s resembles an embedding shaped by its surrounding context. At residue-pair level, z correlates directly with interresidue distance, unlike $s\_\mathrm{input}s^{2}$ and $s^{2}$. These observations reflect the sequential information flow within Boltz-2. From s_inputs through s, z, and distogram, the representation is progressively compressed (384→128→64) toward the downstream objective of generating coordinates. As this compression proceeds, the representation increasingly reflects spatial distance while shedding other factors.

However, binding affinity depends not only on the geometric arrangement of residues but also on the structurally-contextualized residue information carried by the intermediate representations. Existing models typically rely on residue identity combined with predicted structure or pairwise distances (akin to s_inputs plus distogram), whereas the contextualized representation s sits between these and is generally not accessible to models that use only the final structural output.

Notably, Boltz-2 built its own affinity module based on the penultimate representation rather than the final distogram, and Liu *et al.* (2025) showed that coordinates alone provide limited accuracy of thermodynamic interactions. Furthermore, AlphaFold3 explicitly mention the key limitation of protein structure prediction models as predicting static structures and not the dynamic behavior (Abramson *et al.* 2024). We do not claim that later-stage representations are uninformative, but the dimensionality trend and PCA observations together suggest that relying exclusively on the final compressed representations risks discarding the rich information relevant to binding affinity.

### 3.5 Case study on CR6261 antibody

To validate PreFold-dG on naturally evolved, multi-point mutations, we selected the CR6261–H1 antibody–antigen dataset from Phillips *et al.* (2021), who measured dissociation constants $K_{D}$ for combinatorial variants of somatic mutations across CDR and framework regions of a broadly neutralizing anti-influenza antibody. This subset encompasses 11 mutational sites (2048 possible combinations), of which 1844 have experimentally determined $K_{D}$ values. We trained PreFold-dG on SKEMPI except for hemagglutinins (Table S10) detected from measuring homologies (Fig. S1). PreFold-dG predicted ΔG values on this held-out data, obtaining SRCC of 0.725, PCC of 0.682, and MAE of 2.190.

ESM-IF1 is an inverse folding model whose log-likelihood has been used as an unsupervised proxy for binding affinity ranking. Its SRCC of 0.58 on CR6261-H1 (Shanker *et al.* 2024) provides a baseline for what protein structure alone can recover. PreFold-dG achieves SRCC 0.725 on the same dataset after excluding all SKEMPI complexes with sequence homology to H1/H9, a + 25% relative improvement over this baseline. The gain reflects the additional information available from ΔG-supervision over unsupervised structure-aware methods.

**Figure 3:**
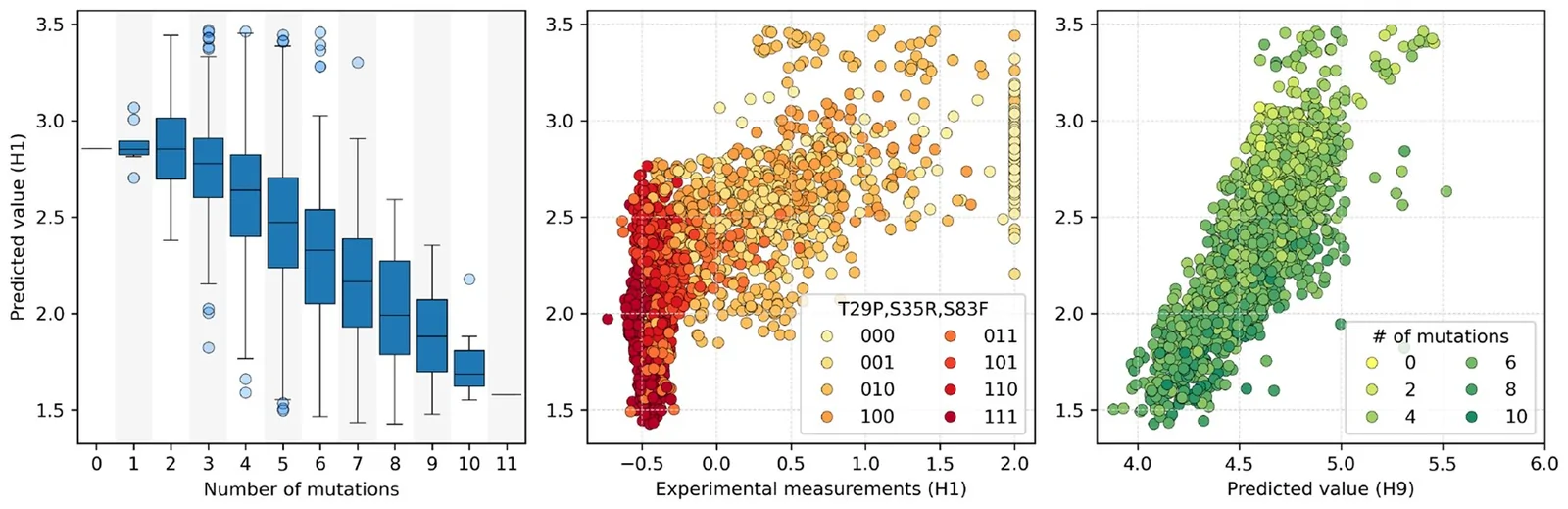
Case study on the CR6261 antibody. (Left) Predicted ΔG stratified by the number of mutations relative to the germline. The overall increase with an initial decrease in affinity, captures the epistasic tendency reported. (Center) Predicted versus experimental ΔG, grouped by the mutational states of three cooperatively interacting residues (T29P, S35R, S83F). (Right) Predicted ΔG of the same CR6261 genotypes binding to influenza H1 and H9, reproducing the pleiotropic correlation observed.

We further examined whether the predictions recapitulate key biological findings of the original study (Fig. 3). The left panel displays predicted ΔG values stratified by mutation count relative to the germline. The mean predicted ΔG exhibits a non-monotonic trend, with an initial increase in binding energy (decrease in binding affinity) at low mutation counts followed by gradual recovery as further mutations accumulate. This is consistent with the epistasis reported, where only one out of the eleven mutations was independently favorable for H1 binding.

Among the selected mutations, T29P, S35R, and S83F were reported to exhibit cooperative behavior in binding. The center panel shows that pairwise combinations (11,01,01,011) predominantly display stronger bindings than single mutants (10,00,10,001), while the additional gain from triple mutant (111) is marginal, reflecting “diminishing returns” from Phillips *et al.* (2021). The pattern is observed in both experimental and predicted values. This shows that the intermediate embeddings may implicitly encode spatial relationships relevant to such cooperative effects, although disentangling structural encoding from statistical regularities requires further investigation.

Finally, predictions of PreFold-dG for the same CR6261 genotypes binding to influenza H1 and H9 are strongly correlated (SRCC: 0.786; PCC: 0.796). While this partly reflects the evident structural similarity between H1 and H9 stem epitopes, the ability to capture the pleiotropic patterns on combinatorial data without fine-tuning is encouraging. Altogether, these results point to the potential of computational approaches for exploring binding affinity landscapes across diverse evolutionary and functional contexts, and motivate further validation on a broader set of antibody–antigen systems.

## 4 Conclusion

Proteins serve as fundamental units of biological function, and protein–protein binding underpins a vast range of cellular processes. Accurate modeling of binding affinity therefore holds significance not only for advancing our understanding of molecular interactions, but also for practical applications such as therapeutic antibody design (Bang *et al.* 2025).

In this study, we present PreFold-dG, a binding affinity model that leverages intermediate representations from Boltz-2. By aggregating these embeddings into compact representations via distance-weighted pooling and decomposing ΔΔG prediction into independent ΔG estimation, PreFold-dG achieves SOTA performance on major benchmarks. We further demonstrated its biological relevance by reproducing epistatic and pleiotropic patterns observed in CR6261, whose mutations have more evolutionary relevance. We hope this work extends to other properties such as stability prediction, and encourages further exploration at the intersection of protein structure prediction and biophysical property modeling.

## Author contributions

Sungjoon Park (Conceptualization, Software, Writing—original draft), Soorin Yim (Investigation, Validation), Dongyun Kim (Methodology) Kiwoong Yoo (Investigation), Doyeong Hwang (Data curation), Kyungwook Lee (Data Curation), Jongseong Jang (Funding acquisition), and Kiyoung Kim (Project administration, Supervision, Writing—review & editing)

## Supplementary Material

btag489_Supplementary_Data

## Data Availability

The data and code underlying this article are available at https://github.com/LGAI-Research/PreFold-dG.
