# Supplementary material for: PreFold-dG: estimating binding affinity of protein–protein interaction from intermediate representations of protein folding model
Source: Bioinformatics. 2026 Aug 21;42(Suppl 2):btag489. doi: 10.1093/bioinformatics/btag489 (PMC13501319; doi:10.1093/bioinformatics/btag489)
Supplement: Supplementary file 1 [file btag489_supplementary_data.pdf]

A. Supplementary Materials

A.1. Supplementary Figures

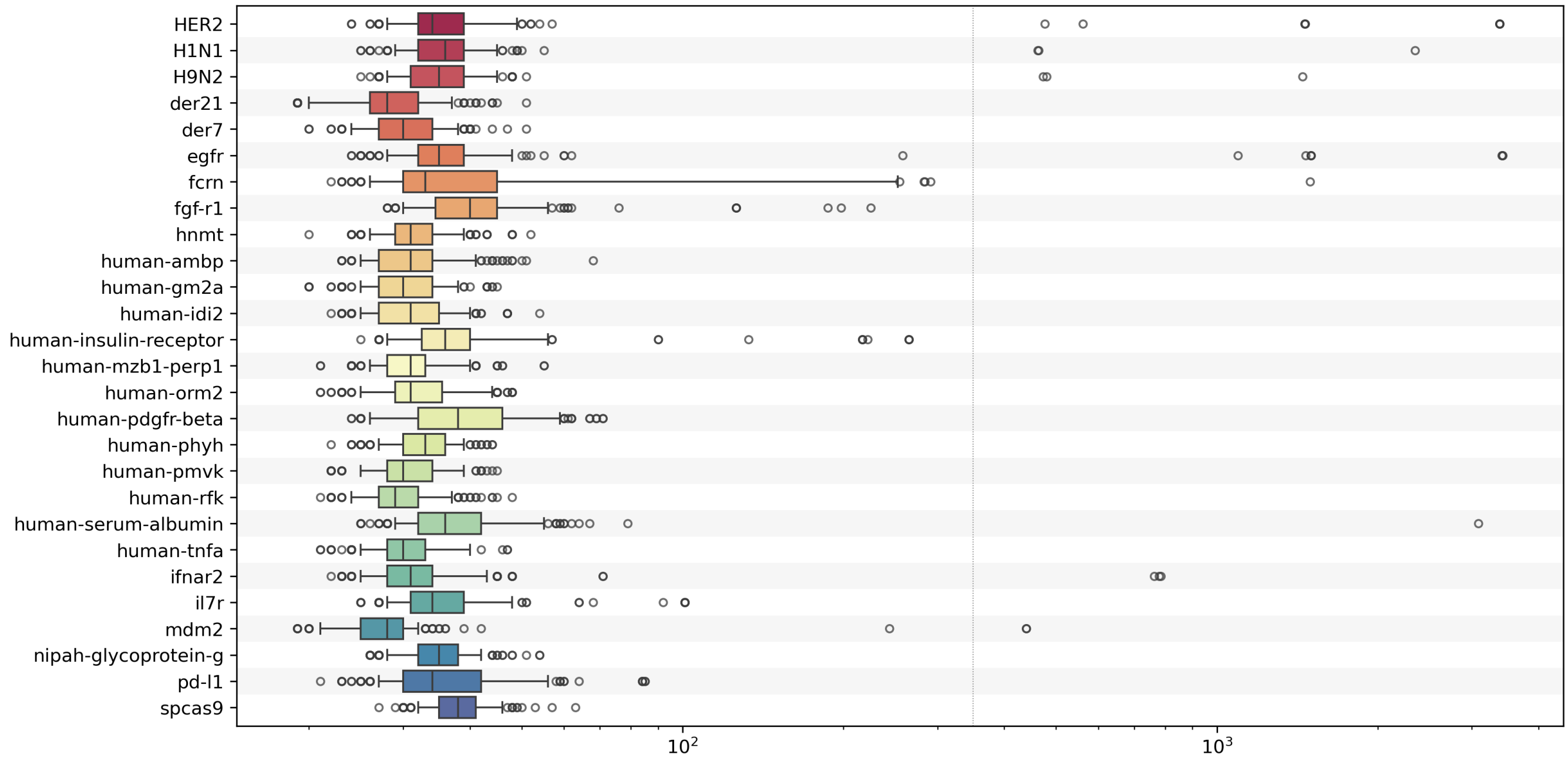

Figure S1 SKEMPI distribution of local sequence alignment scores against various target proteins. To prevent structural information leakage when evaluating external protein datasets such as HER2 binders (HER2), influenza hemagglutinins (H1N1 and H9N2), and Adaptyv competition data (the rest), homology to each target was measured using the Smith-Waterman algorithm with the BLOSUM62 similarity matrix and gap penalties of -12 (opening) and -2 (extension). Whiskers span the 5th to 95th percentiles. Proteins were considered homologous if their alignment score exceeded 350. Inference on each target was performed using PreFold-dG trained on SKEMPI with homologous proteins excluded.

A.2. Supplementary Tables

Table S1. Stratification of binding affinity measurement methods in SKEMPI. Tiers are assigned heuristically based on the reliability and abundance in the dataset.

Tier	Methods[1]
1	SPR, ITC, BLI, KinExA
2	CSPRIA, ELISA, ELFA, SFFL, SFPP, SE, SP, RA, ESMA, IARA, IAGE, IAFL
3	IASP, EMSA, FL

[1]See https://life.bsc.es/pid/skempi2/database/summary for methods details.

Table S2. Hyperparameters used to produce the results of this paper.

PreFold-dG

Phase	Hyperparameter	Value
Model	s_inputs_encoder_hidden_dim	198
	s_inputs_encoder_dropout	0.25
	s_encoder_hidden_dim	198
	s_encoder_dropout	0.25
	z_encoder_dropout	0.1
	embed_dim	24
	predictor_hidden_dim	32
Pre-processing	interface_threshold	12[1]
Experiments	batch_size	64
	epochs	50
	learning_rate	.0001
	weight_decay	.0001

[1]If the 1st percentile of interchain distances is larger than 12, we take that value as the threshold of interface.

Boltz-2

Type	Hyperparameter	Value
Integer	seed	42
	recycling_steps	10
	diffusion_samples	1
	max_parallel_samples	1
	max_msa_seqs	16,384
String	checkpoint	`boltz2`
Boolean	use_potentials	`True`
	use_msa_server	`True`
	write_full_pde	`True`
	write_full_pae	`True`
	write_pae_logits	`True`
	write_conf_inputs	`True`

Table S3. More table for $\Delta\Delta G$ evaluation on the SKEMPI dataset using the 3-fold cross-validation split of RDE-Network.

Method	Per-structure		Overall				
	PCC ↑	SRCC ↑	PCC ↑	SRCC ↑	RMSE ↓	MAE ↓	AUROC ↑
MIF-Network	0.3965	0.3509	0.6523	0.5134	1.5932	1.1469	0.7329
DDGPred	0.3750	0.3407	0.6580	0.4687	1.4998	1.0821	0.6992
End-to-End	0.3873	0.3587	0.6373	0.4882	1.6198	1.1761	0.7172
DiffAffinity	0.4220	0.3970	0.6690	0.5560	1.5350	1.0930	0.7440
Surface-VQMAE	0.4694	0.4324	0.6482	0.5611	1.5876	1.1271	0.7469
ProMIM	0.4640	0.4310	0.6720	0.5730	1.5160	1.0890	0.7600
GeoAB	0.3936	0.3610	0.6324	0.4972	1.4758	1.0611	N/A
Prompt-DDG	0.4712	0.4257	0.6772	0.5910	1.5207	1.0770	0.7568
Pi-SAGE	0.4230	0.3450	0.6000	0.4280	1.8170	1.3060	0.6910
CATH-ddG	0.4600	0.5251	0.7151	0.6393	1.4447	1.0335	0.7760
Refine-PPI	0.4561	0.4374	0.6592	0.5608	1.5643	1.1093	0.7542
EnerBridge-DPO	0.4981	0.4666	0.7487	0.6447	1.4257	1.0185	0.7780
PreFold-dG	0.6109	0.5732	0.7500	0.6597	1.6023	1.1292	0.7901

Table S4. PDB structures from SKEMPI with homology to HER2 visualized using PyMOL. For each complex, the HER2 extracellular domain is shown in red and its binding partner in blue. All structures are superimposed on either chain to illustrate structural similarity.

PDB: `1N8Z`	PDB: `3BE1`	PDB: `3N85`
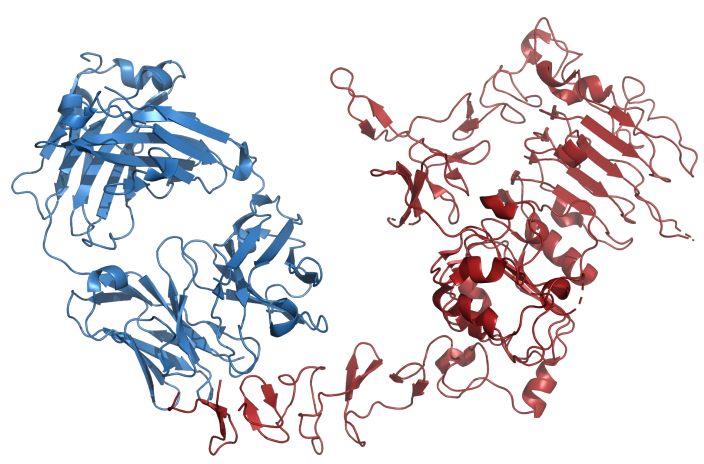	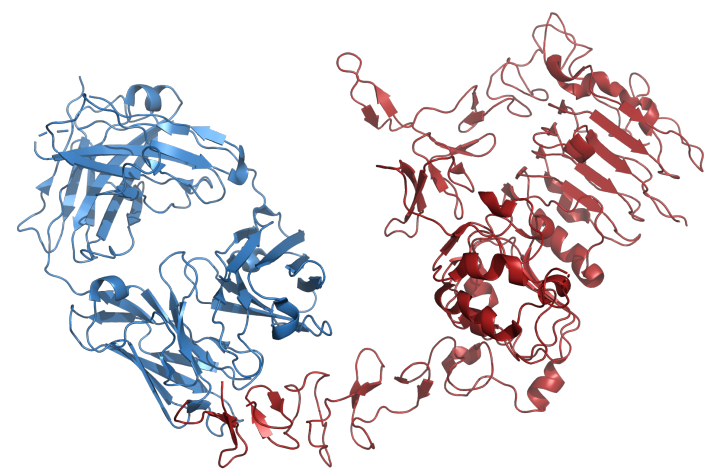	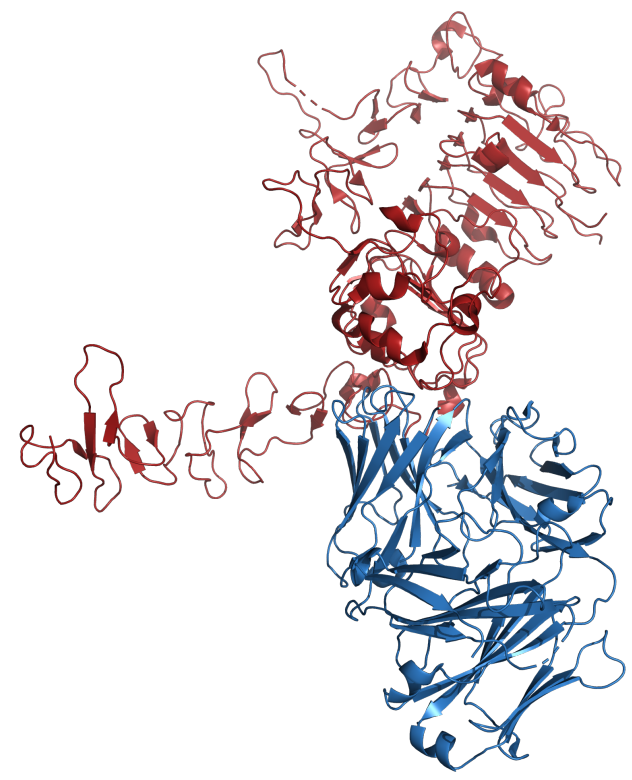
PDB: `4KRL`	PDB: `3MZW`	PDB: `4HRN`
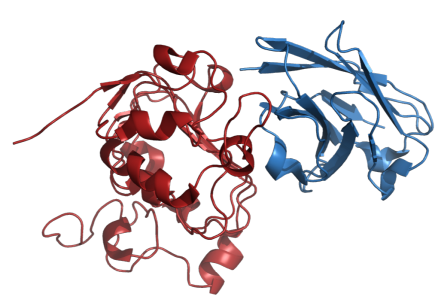	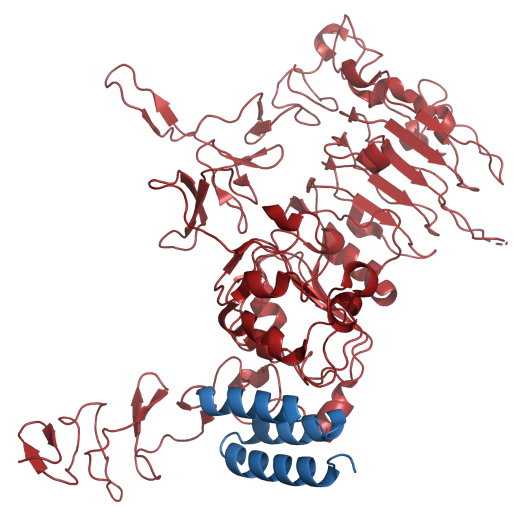	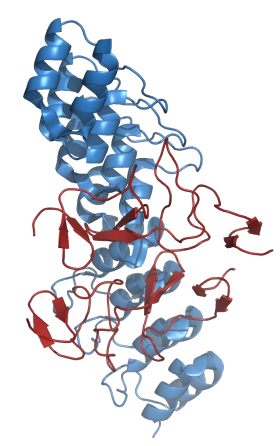
PDB: `1YY9`	PDB: `4KRO`	PDB: `4KRP`
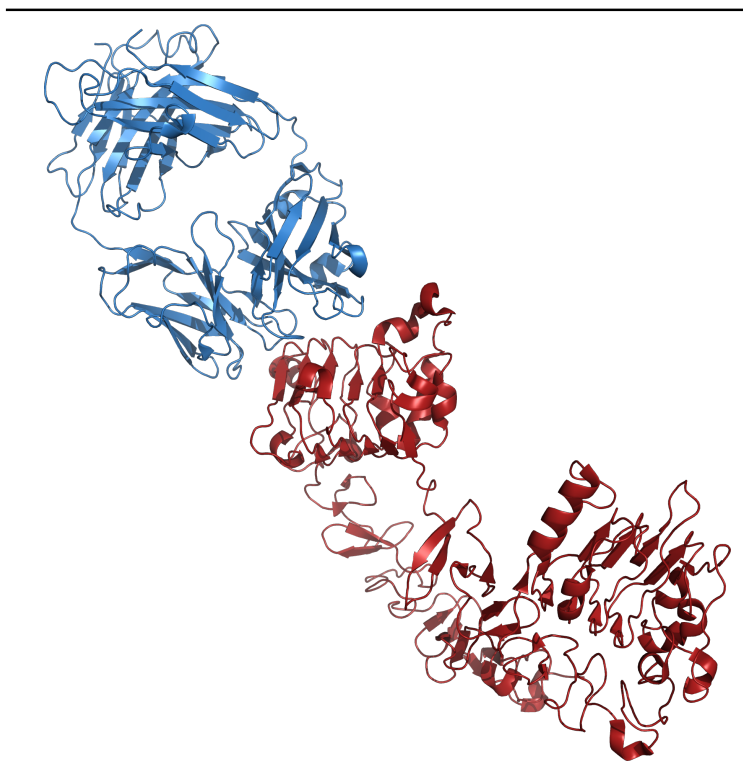	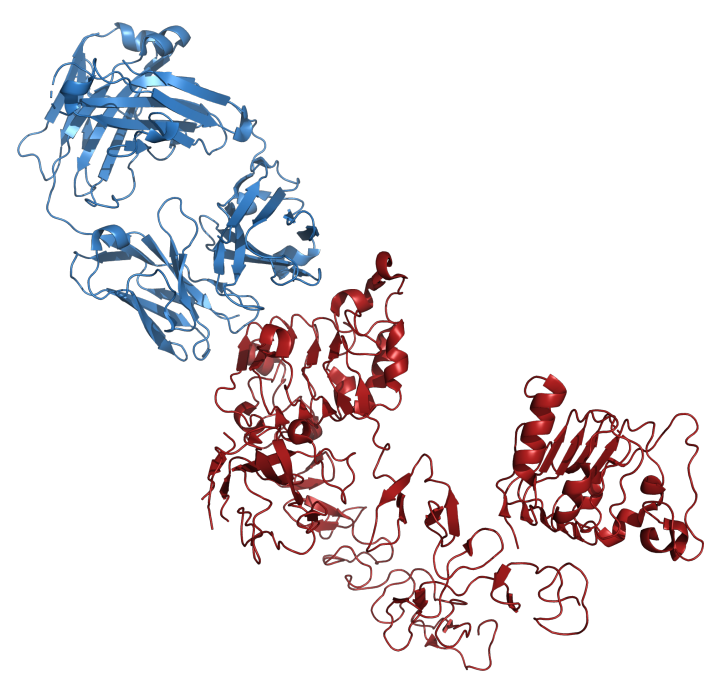	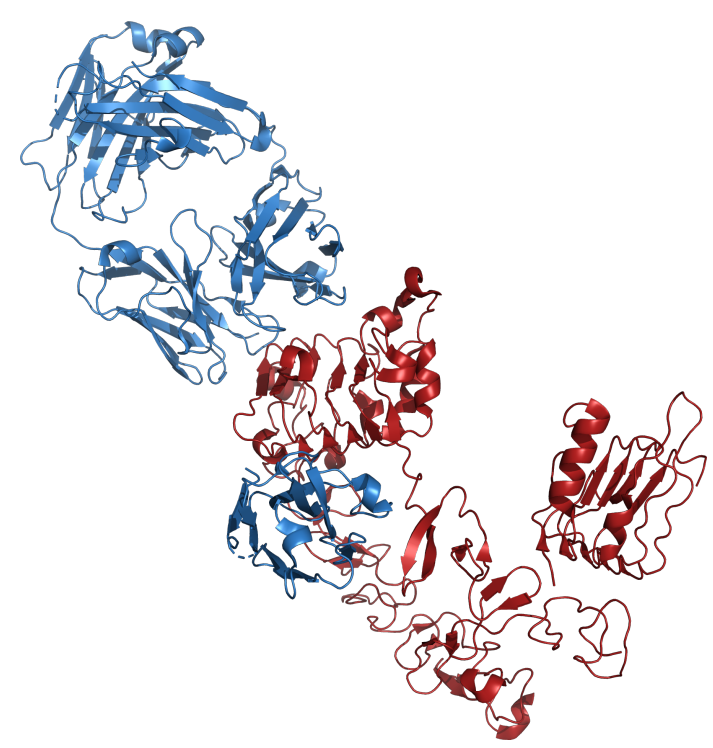

Table S5. $\Delta\Delta G$ evaluation on the HER2 dataset using the 5-fold cross-validation split of GearBind. Predicted $\Delta\Delta G$ values are computed by averaging across models trained on each of the five folds.

Metric	FoldX	Flex-ddG	Bind-ddG	GearBind+P	PreFold-dG
PCC ↑	0.246	0.387	0.478	0.515	0.524
SRCC ↑	0.329	0.388	0.475	0.519	0.529

Table S6. Per-fold $\Delta\Delta G$ evaluation (SRCC) on the HER2 dataset using the split of GearBind. The per-fold predictions reported for GearBind are from the model without pretraining, as only those results are publicly available[1]. The pretrained version (denoted GearBind+P in the original paper) may exhibit less per-fold variance.

Method	Fold 0	Fold 1	Fold 2	Fold 3	Fold 4	Mean[2]
GearBind	0.350	0.291	0.486	0.533	0.376	0.475
PreFold-dG	0.520	0.523	0.532	0.474	0.507	0.543

[1]`https://github.com/DeepGraphLearning/GearBind/blob/main/results/GearBind_HER2_1n8z_renum.pdb_HL_C.csv`

[2]Evaluation of SRCC score using the mean ΔG values of all five folds.

Table S7. Assessment on the Adaptyv competition data[1], which provides experimental ΔG measurements for computationally designed proteins. Expressed proteins labeled as either binding (B) or non-binding (NB) were evaluated as a classification task. Among these, binding proteins were additionally annotated with K_D values, which we evaluated directly as a regression task. The final row (Total) reports the summed protein count and the macro-averaged value for each metric.

Target	Counts		Classification [NB+B]			Regression [B]			
	NB	B	ACC[2] ↑	AUROC ↑	AUPRC ↑	SRCC ↑	PCC ↑	RMSE ↓	MAE ↓
der21	12	4	0.7500	0.8125	0.9409	0.6000	0.4067	1.0163	0.7386
der7	15	5	0.7500	0.7467	0.9257	0.2000	0.3034	1.3944	1.3663
egfr	634	134	0.7656	0.6859	0.9099	0.2109	0.2310	1.9931	1.7343
fcrn	17	6	0.6957	0.3824	0.6543	-0.1429	-0.0375	5.6982	5.4924
fgf-r1	10	7	0.5294	0.4714	0.6514	0.4643	0.1430	3.7925	3.7222
hnmt	26	2	0.6071	0.7885	0.9799	-1.0000	-1.0000	3.3511	3.1464
human-ambp	25	5	0.6000	0.6720	0.9307	0.0000	0.0901	3.8026	3.7590
human-gm2a	25	0	N/A	N/A	N/A	N/A	N/A	N/A	N/A
human-idi2	18	5	0.3913	0.5556	0.8474	0.1000	0.3729	3.7575	3.7114
human-insulin-receptor	14	21	0.6857	0.6939	0.4871	-0.1818	-0.1939	1.4243	1.1670
human-mzb1-perp1	15	15	0.8333	0.7556	0.6504	-0.1214	0.0012	4.3645	4.2765
human-orm2	23	0	N/A	N/A	N/A	N/A	N/A	N/A	N/A
human-pdgfr-beta	12	18	0.6000	0.5139	0.3658	-0.4241	-0.4075	3.8809	3.5861
human-phyh	6	6	0.5000	0.3056	0.3713	-0.0286	0.0173	3.9311	3.8696
human-pmvk	19	10	0.7241	0.7368	0.8666	0.5879	0.6327	4.3901	4.3536
human-rfk	22	2	0.9167	0.9545	0.9959	1.0000	1.0000	2.8645	2.8591
human-serum-albumin	103	0	N/A	N/A	N/A	N/A	N/A	N/A	N/A
human-tnfa	32	0	N/A	N/A	N/A	N/A	N/A	N/A	N/A
ifnar2	12	8	0.6500	0.5729	0.7220	0.5000	0.2841	3.3401	3.2946
il7r	35	62	0.4433	0.5088	0.4225	-0.0465	-0.0164	4.1108	3.9869
mdm2	15	12	0.6667	0.5944	0.6116	0.1190	0.6082	3.5973	3.2917
nipah-glycoprotein-g	781	103	0.5950	0.5906	0.9170	0.2694	0.1890	2.8888	2.7220
pd-l1	36	39	0.7067	0.7179	0.6503	0.1032	0.0334	1.6102	1.1222
spcas9	4	14	0.8333	0.8036	0.3693	-0.1538	-0.0701	1.8040	1.7073
Total	1,911	478	0.6622	0.6432	0.7135	0.1028	0.1294	3.1506	2.9954

[1]`https://proteinbase.com/proteins`

[2]Accuracy (ACC) was computed using the optimal threshold derived from the AUROC curve, defined as the threshold that maximizes the true positive rate minus the false positive rate.

Table S8. Ablation studies on PreFold-dG, evaluated on the SKEMPI dataset using the 3-fold cross-validation split of RDE-Network. PreFold-dG denotes the full model, while `*−s_inputs`, `*−s`, and `*−z` denote variants with each respective feature ablated. ΔG_{mut} *only* and $\Delta\Delta G$ *only* use each supervision in isolation, rather than the full joint loss. Ablated models show degraded performance across most metrics, suggesting that the removed features capture fine-grained binding signatures rather than coarse structural characteristics, and that the joint loss contributes to training stability.

Method	Per-structure		Overall ($\Delta\Delta G$)			Overall (ΔG)	
	PCC ↑	SRCC ↑	PCC ↑	SRCC ↑	RMSE ↓	PCC ↑	SRCC ↑
PreFold-dG	**0.6109**	**0.5732**	**0.7500**	**0.6597**	1.6023	**0.7114**	**0.7122**
`*−s_inputs`	0.5759	0.5442	0.7330	0.6315	1.5123	0.6630	0.6556
`*−s`	0.5178	0.4827	0.6543	0.5867	1.6848	0.6828	0.6871
`*−z`	0.5881	0.5401	0.7477	0.6327	**1.5080**	0.7099	0.7015
ΔG_{mut} *only*	0.5869	0.5434	0.7090	0.6464	1.6926	0.7090	1.8092
$\Delta\Delta G$ *only*	0.5953	0.5636	0.7392	0.6415	1.5974	0.4982	2.4332

Table S9. Sensitivity analysis of PreFold-dG to the weight of ΔG_{wt} in the joint loss term. 1 (baseline) trains on the wild-type ΔG_{wt} each time its mutant ΔG_{mut} is encountered, weighted equally with ΔG_{mut} and the corresponding $\Delta\Delta G$ term. $1/16$ compensates for this redundancy using the average scale difference between mutant and wild-type occurrences. $1/N$ reweights each wild-type by the inverse of its count N, equalizing the contribution within a batch. $1/\sqrt{N}$ applies more moderate corrections. $16/N^2$, conversely, amplifies the imbalance. The observed effects are marginal, while the baseline remains superior or comparable across all metrics.

ΔG_{wt} weight	Per-structure		Overall ($\Delta\Delta G$)			Overall (ΔG)	
	PCC ↑	SRCC ↑	PCC ↑	SRCC ↑	RMSE ↓	PCC ↑	SRCC ↑
1 (baseline)	**0.6109**	**0.5732**	0.7500	0.6597	1.6023	**0.7114**	**0.7122**
$1/16$	0.6059	0.5688	**0.7528**	0.6574	**1.5610**	0.6710	0.6630
$1/\sqrt{N}$	0.5979	0.5578	0.7502	0.6593	1.5640	0.6842	0.6675
$1/N$	0.6021	0.5637	**0.7528**	**0.6601**	1.5647	0.6879	0.6811
$16/N^2$	0.6092	0.5703	0.7395	0.6457	1.5720	0.6917	0.6885

Table S10. PDB structures from SKEMPI with homology to H1N1 visualized using PyMOL. For each complex, the hemagglutinin is shown in red and its binding partner in blue. All structures are superimposed on the hemagglutinin to illustrate structural similarity.

PDB: `3LZF`	PDB: `2VIR`	PDB: `2VIS`
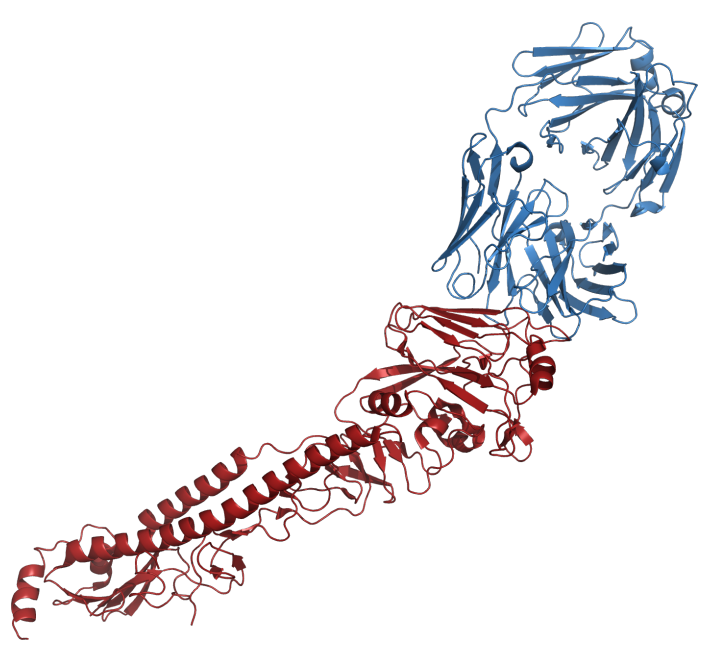	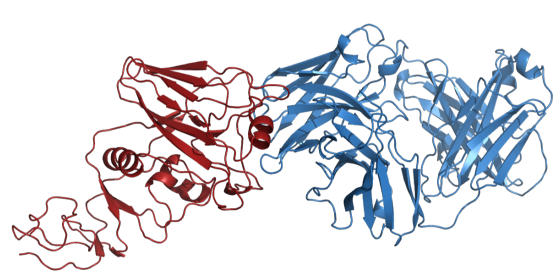	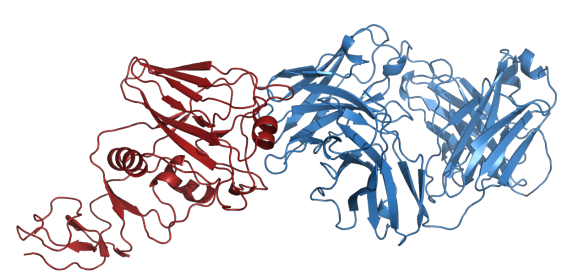
